# The Impact of Background-Level Carboxylated Single-Walled Carbon Nanotubes (SWCNTs−COOH) on Induced Toxicity in *Caenorhabditis elegans* and Human Cells

**DOI:** 10.3390/ijerph19031218

**Published:** 2022-01-22

**Authors:** Jian-He Lu, Wen-Che Hou, Ming-Hsien Tsai, Yu-Ting Chang, How-Ran Chao

**Affiliations:** 1 Emerging Compounds Research Center, General Research Service Center, National Pingtung University of Science and Technology, Pingtung 912, Taiwan; toddherpuma@mail.npust.edu.tw (J.-H.L.); hrchao@mail.npust.edu.tw (H.-R.C.); 2 Department of Environmental Engineering, National Cheng Kung University, Tainan 701, Taiwan; 3 Department of Child Care, College of Humanities and Social Sciences, National Pingtung University of Science and Technology, Pingtung 912, Taiwan; alantsai@mail.npust.edu.tw; 4 Research Institute for Life Support Innovation, Research Organization for Nano & Life Innovation, Waseda University, 2-2 Wakamatsucho, Shinjuku, Tokyo 162-8480, Japan; 5 Department of Environmental Science and Engineering, College of Engineering, National Pingtung University of Science and Technology, Pingtung 912, Taiwan; b22572193@gmail.com; 6 Institute of Food Safety Management, College of Agriculture, National Pingtung University of Science and Technology, Pingtung 912, Taiwan; 7 School of Dentistry, College of Dental Medicine, Kaohsiung Medical University, Kaohsiung 807, Taiwan

**Keywords:** *Caenorhabditis elegans*, carboxylated single-walled carbon nanotubes, nanotoxicity, in vivo, environment

## Abstract

Single-walled carbon nanotubes (SWCNTs) are widely utilized for industrial, biomedical, and environmental purposes. The toxicity of Carboxylated SWCNTs (SWCNTs−COOH) in in vivo models, particularly *Caenorhabditis elegans* (*C. elegans*), and in vitro human cells is still unclear. In this study, *C. elegans* was used to study the effects of SWCNTs−COOH on lethality, lifespan, growth, reproduction, locomotion, reactive oxygen species (ROS) generation, and the antioxidant system. Our data show that exposure to ≥1 μg·L^−1^ SWCNTs−COOH could induce toxicity in nematodes that affects lifespan, growth, reproduction, and locomotion behavior. Moreover, the exposure of nematodes to SWCNTs−COOH induced ROS generation and the alteration of antioxidant gene expression. SWCNTs−COOH induced nanotoxic effects at low dose of 0.100 or 1.00 μg·L^−1^, particularly for the expression of antioxidants (SOD-3, CTL-2 and CYP-35A2). Similar nanotoxic effects were found in human cells. A low dose of SWCNTs−COOH induced ROS generation and increased the expression of catalase, MnSOD, CuZnSOD, and SOD-2 mRNA but decreased the expression of GPX-2 and GPX-3 mRNA in human monocytes. These findings reveal that background-level SWCNTs−COOH exerts obvious adverse effects, and *C. elegans* is a sensitive in vivo model that can be used for the biological evaluation of the toxicity of nanomaterials.

## 1. Introduction

Engineered nanomaterials (ENMs), which are man-made materials with at least one dimension in the size range of 1–100 nm, have attracted strong interest for research and real-world applications in industrial processes and consumer products. Due to their widespread use, ENMs are inevitably released into the environment. This has prompted great scientific and public concern as to the ecological and human health risks resulting from exposure to ENMs [1]. In particular, single-walled carbon nanotubes (SWCNTs) are known to exhibit attractive mechanical, magnetic, optical, thermal, chemical, surface, and, most importantly, electric properties that surpass those of other carbon nanotube (CNT) variants [2,3,4]. Ever since their discovery by Iijima and Ichihashi [5], SWCNTs have been utilized for industrial, biomedical, and environmental purposes, such as for drug delivery, bioimaging, sensors, nanoelectrodes, lithium-battery anodes, supercapacitors, and electron field emitters as well as catalysts, polymer additives, and reinforcements in nanocomposites [6,7,8,9]. However, the poor water solubility and tendency towards aggregation of unfunctionalized CNTs have hindered their determination and characterization in aqueous environments [10]. In addition, CNTs are known to be resilient to degradation and may persist for an unknown period of time in the environment [11,12]. Therefore, the standard quantification of CNT concentrations in different environmental samples remains a challenge [13].

CNTs are entirely composed of a priori carbon atoms that are generally considered to be of low toxicity but which have been classified as “emerging pollutants” by the U.S. Environmental Protection Agency due to their highly toxic effects on cells and organisms [14,15,16]. Concern about the potential adverse effects of human exposure to CNTs during their manufacture, usage, and disposal has increased [17]. The toxicity of CNTs is mainly attributed to their physiochemical properties, such as their nanoscale size and fibrous structure (which can exhibit asbestos-like toxicity) [18]. Chemical modifications can improve the dispersion of SWCNTs and modify the toxicological effects, but the introduction of functional groups may also raise the level of toxicity [19]. Carboxylation is the most common strategy used to improve aggregation and water solubility and increase the usage and further functionalization of CNTs via carboxyl groups [20]. In addition, carboxylated SWCNTs (SWCNTs−COOH) also protect SWCNTs from photo-induced aggregation via scavenged OH during synthesis [21]. However, SWCNTs−COOH has been observed to be more toxic than SWCNTs, SWCNTs-CONH_2_, and SWCNTs-PEG [22]. In vitro studies have reported the cytotoxic effects of SWCNTs−COOH in normal rat kidney (NRK) cells [23] and in multipotent mesenchymal stem cells (MSCs) [24]. Although SWCNTs were not shown to cause cytotoxicity in a human alveolar epithelial cell line (A549), their size and bundle length resulted in a significant change in the generation of intracellular reactive oxygen species (ROS) [25]. Furthermore, SWCNTs−COOH also disturbs gene and protein expression and protein phosphorylation, leading to DNA damage, cell cycle arrest, and genotoxicity [26,27]. In in vivo studies, SWCNTs were observed to induce granulomas in rats [28,29]. Zhang et al. found that the exposure of C57BL/6 mice to SWCNTs−COOH led to acute lung injury via pro-inflammatory cytokine storm signaling regulated by the NF-κB pathway [30]. Aside from pulmonary toxicity, SWCNTs−COOH nanomaterials were observed to decrease the body length and reproduction of daphnids [22].

The nematode of *Caenorhabditis elegans* (*C. elegans*) had been established as a non-mammalian model animal [31]. Due to their inexpensive and facile maintenance, short lifespan, rapid reproduction cycles, high degree of molecular conservation, well-described genetic and genomic tools, simple nervous system, and sensitivity to environmental toxicants, *C. elegans* has been used in both biomedical and environmental toxicity testing by observing several parameters, e.g., lethality, growth kinetics, reproduction, locomotion properties, and reactive oxygen (ROS) production [31,32,33,34,35,36]. Moreover, the *C. elegans* model lacks the animal ethics issues that are present with mammalian animal models. In our previous studies [36,37,38,39], shortened longevity, disruption of locomotion and reproduction, and the induction of an oxidative stress response were found after the nematodes were exposed to PM2.5, graphene oxide nanoparticles, and hydroxyl- and amine-functionalized silica nanoparticles. In *C. elegans*, exposure to multi-walled carbon nanotubes (MWCNTs) not only caused functional damage to both primary (e.g., intestine) and secondary (e.g., reproductive and neurons organs) targeted organs [40,41,42], they also induced translocation from the intestines to secondary targeted organs [40,41]. Previous studies utilizing *C. elegans* for the evaluation of CNT toxicity have reported varying toxicological endpoint results, particularly for the different variants of SWCNTs [43,44]. No significant toxicity was observed in *C. elegans* after exposure to the higher concentration (500 μg/mL) of SWCNTs, but exposure to 500 μg·mL^−1^ of amide-functionalized SWCNTs was associated with a decreased lifespan, reduced body length, and defective embryogenesis of *C. elegans* [43]. Short and long-term exposure to cysteine-functionalized SWCNTs was reported to have no effect on the lifespan, reproduction, and locomotion of *C. elegans* at concentrations of 50, 100, and 250 μg·mL^−1^ [44]. However, the toxicological effects of SWCNTs−COOH on *C. elegans* are still unclear.

Notwithstanding the extensive application of nanomaterials, little is known regarding the impacts of these materials on animal and human health. Therefore, the present study was aimed at investigating the possible toxicity of SWCNTs−COOH using in vitro cultured human cells and an in vivo wild-type *C. elegans* model. The different toxicological endpoints of *C. elegans* were investigated, including physical observations, distribution, translocation, co-localization with ROS, and antioxidant and oxidative stress responses. Finally, we also evaluated the immune response of SWCNTs−COOH on the human monocytic cell line, THP-1. The results from this study may contribute to the existing body of global data on in vivo nanotoxicity testing of the SWCNTs variants.

## 2. Materials and Methods

### 2.1. Reagents

SWCNTs−COOH (catalog no. P3-SWNT) nanomaterials were purchased from Carbon Solutions, Inc. (Riverside, CA, USA). SWCNTs−COOH nanomaterials were synthesized using electric arc discharge and then purified and functionalized with the –COOH group (1.0–3.0 atom % carboxyl groups and >90% (*w*/*w*) SWCNTs), in accordance with the manufacturer. Wild-type N2 nematodes were acquired from the Department of Biochemistry and Molecular Biology, College of Medicine, National Cheng Kung University (Tainan, Taiwan, China). The nematode growth medium (NGM) plates contained bacteriological agar (Laboratories Conda, S.A., Madrid, Spain), bactopeptone (Laboratories Conda, S.A., Madrid, Spain), and NaCl (Honeywell Fluka™, Morris Plains, NJ, USA). OP50 *E. coli* cultures were acquired from the Bioresources Collection and Research Center (Hsinchu, Taiwan, China), and Luria-Bertani broth was obtained from Sigma-Aldrich (St. Louis, MO, USA). The bleaching solution contained NaOCl (J.T. Baker, Central Valley, PA, USA) and KOH (Duksan Pure Chemicals, Ulsan, Gyeonggi-do, South Korea). K-medium (2.36 g KCl (Avantor Performance Materials, Ltd., Suwon, Gyeonggi-do, South Korea), 3 g NaCl, in 1 L ddH_2_O) and M9 buffer solution (7.56 g Na_2_HPO_4_ (Honeywell Fluka™, Charlotte, NJ, USA), 1.5 g KH_2_PO_4_ (Avantor Performance Materials, LLC, Radnor, PA, USA), 2.5 g NaCl, 0.5 mL 1 M MgSO_4_ (Avantor Performance Materials, Ltd.), 500 mL H_2_O) were also prepared in this study. Human monocytic THP-1 cells were purchased from the American Type Culture Collection (ATCC, Rockville, MD, USA). THP-1 cell culture medium containing RPMI-1640 cell culture medium, 10% fetal bovine serum (FBS), 2 mM L-glutamine, 1 mM sodium pyruvate, 10 mM HEPES, 100 U mL^−1^ penicillin, and 100 µg·mL^−1^ streptomycin were all purchased from Gibco, Life Technologies (Carlsbad, CA, USA). Dehydroethidium (DHE) and CellROX™ Green Reagent for oxidative stress detection were purchased from Invitrogen™, Thermo Fisher Scientific (Waltham, MA USA). TRIzol Reagent, the High-Capacity cDNA Reverse Transcription kit, SYBR green PCR master mix, and Alamar Blue reagent were also purchased from Gibco, Life Technologies (Carlsbad, CA, USA). All physiological observations were done under a dissecting microscope (Olympus, SZX10, Waltham, MA, USA).

### 2.2. Characterization of SWCNTs−COOH

Characterization was performed in the Instrument Center of National Cheng Kung University. The morphology of SWCNTs−COOH in K-medium was examined using a JEOL JEM-7000F transmission electron microscopy (TEM). An elemental analysis was conducted using energy-dispersive X-ray spectroscopy (EDS). For the TEM and EDS analyses, aliquots of the sample were dropped on copper mesh and air-dried at room temperature. Fourier-transform infrared spectroscopy (FTIR) results are cited from our earlier study using the same SWCNTs−COOH sample [21].

### 2.3. C. elegans Maintenance, Acute Exposure Treatment, Lifespan, and Lethality Assays of C. elegans

N2 nematodes were maintained on OP50 *E. coli*-seeded nematode growth medium (NGM) plates at 22 °C. Synchronization of N2 cultivated nematodes was done using the alkaline bleaching method, as described previously [45]. After growth from the synchronized L1 nematodes, the L3 or young·L4 nematodes were rinsed from the plate with K-medium and centrifuged at 2500× *g* for 4 min. The worm pellet was diluted with K-medium, and approximately 200 L3/young·L4 nematodes were dispensed in each well of the 12-well plate. The SWCNTs−COOH were pre-dispersed in K medium by sonication for 30 min (40 kHz, 100 W). After starvation for 24 h, the nematodes were exposed to 0, 0.001, 0.01, 0.1, 1, 10, 100, or 1000 μg·L^−1^ SWCNTs−COOH for 24 h (acute exposure), and then 50 worms were transferred onto NGM plates without OP50. The nematodes were gently prodded using the worm picker to assess their viability. Worms that were non-responsive to a stimulus were considered dead. For the lethality assay, the N2 wild type strain nematodes were transferred to fresh NGM plates after acute exposure and scored as dead or alive, while for the lifespan assay, the nematodes were transferred every day to fresh NGM plates, and their survival was scored for a duration of 24 days. These assays were done in triplicate for each concentration.

### 2.4. Growth Measurement of C. elegans

The current study measured the lengths of nematodes to assess the effects of SWCNTs−COOH on growth. After exposure to the different concentrations of SWCNTs−COOH for 24 h, the nematodes were transferred to NGM plates with OP50 lawn and incubated for 48 h until the old L4 stage. An Olympus SZX10 dissecting microscope was used to obtain images, while ImageJ software [46] was used to measure the length of each of the nematodes. Three worms were measured for each concentration.

### 2.5. Locomotion Assay of C. elegans

Twenty L3/young·L4 nematodes were dispensed in each well of a 12-well plate and starved for 24 h, before undergoing an assessment of their locomotion behavior following exposure to the different concentrations of SWCNTs−COOH for 24 h. Head thrashing and body bending were used to assess the locomotion behavior of *C. elegans*. The evaluation of head thrashing and body bending was described in a previous study [41]. Head thrashing was defined as a change in the direction of mid-body bending, while body bending was described as a direction change of the nematode part corresponding to the posterior bulb of the pharynx with respect to the *y* axis while the nematode was traveling along the *x* axis. Head thrashing was scored for 1 min, while body bends were counted in an interval of 20 s. Three replicates were performed for each concentration.

### 2.6. Reproductive Assay of C. elegans

Following acute exposure, L3/young·L4 nematodes were assessed for 4 to 5 days of egg-laying. One L3/young·L4 nematode was dispensed in each well of a 12-well NGM plate with fresh OP50 lawn. Each nematode was transferred to a new plate every 2 days during spawning. The old plates containing the eggs were hatched and incubated to L4 nematodes to make it easier to count offspring. The total number of offspring for each of the worms was counted. Twelve worms were evaluated per treatment group.

### 2.7. Intracellular ROS Staining

The N2 nematodes were exposed as described above. After acute exposure, the worms were transferred and maintained in fresh NGM plates for 8 days (based on what day a surviving population of almost 50% was observed). Therefore, the intracellular ROS level of the nematodes was evaluated after 8 days of exposure to SWCNTs−COOH at all concentrations. The examined nematodes were exposed to 5 μM of dehydroethidium (DHE) dye and incubated at room temperature in the absence of light for 30 min. The supernatant was discarded afterwards. An Olympus FV1000 laser-scanning confocal microscope was used to determine the ROS levels.

### 2.8. Intracellular ROS Localization Assays of C. elegans

A laser-scanning confocal microscope was used to detect the SWCNTs−COOH nanomaterials conjugated with Rhodamine B [44]. Rho B was adsorbed on the SWCNTs−COOH surface to track its distribution in the nematodes. The preparation of Rhodamine B-conjugated SWCNTs−COOH was described previously [41]. Rho B-conjugated SWCNTs−COOH nanomaterials were prepared by mixing Rho B solution (1 mg·mL^−1^, 0.3 mL) with an aqueous suspension of SWCNTs−COOH (0.1 mg·mL^−1^, 5 mL). The mixture solution was then subjected to dialysis against distilled water for 72 h to remove unbound Rho B. The nematodes were exposed to 1000 μg/L of SWCNTs−COOH /Rho B and incubated for 3 h in the absence of light at room temperature. The nematodes were then washed with K medium and exposed to 5 μM of CellROX™ (green) for 30 min for ROS staining. The exposed nematodes were then observed under the laser scanning confocal microscope.

### 2.9. Cell Viability and ROS Generation Assays of THP-1 Cells

The human monocyte cell line THP-1 was cultured at 37 °C (5% CO_2_ with humidity) in RPMI 1640 with 10% FBS and 1% penicillin/streptomycin. The THP-1 cells were resuspended in fresh media at a concentration of 2 × 10^5^ mL^−1^, 0.1 mL per well in 96-well plates and reacted with a 20 mM 2′,7′-dichlorodihydrofluorescein diacetate (DCFH-DA) probe for 30 min. Following treatment with SWCNTs−COOH at all concentrations for 6 h, the fluorescence intensity of the stained cells was measured at 485 nm (excitation) and 527 nm (emission) to determine the ROS level. Cell viability was determined by Alamar Blue assay. The THP-1 cells were resuspended in fresh media at a concentration of 2 × 10^6^ mL^−1^, with 0.1 mL per well in 96-well plates, and reacted with 10% Alamar Blue reagent for 30 min. Following treatment with SWCNTs−COOH at all concentrations for 48 h, the fluorescence intensity of the stained cells was measured at 530–560 nm (excitation) and 590 nm (emission). Data were calculated as the percent (%) relative to the untreated control, which was set at 100%. Three replicates were performed for each of the concentrations.

### 2.10. Quantitative Real-Time PCR (qRT-PCR) Assays

Total RNA from each sample was isolated from *C. elegans* and the THP-1 cells using TRIzol Reagent and reverse-transcribed with the High-Capacity cDNA Reverse Transcription kit. For real-time PCR, cDNA (50 ng) was amplified with SYBR green PCR master mix through a 95 °C denaturation stage for 10 min, followed by 40 repetitions of 95 °C for 15 s and then 60 °C for 1 min using an Applied Biosystems PRISM 7500 fast real-time PCR system. For *C. elegans*, we quantified the expression of superoxide dismutase-1 (SOD-1), SOD-3, ctl-2, mtl-2, cyp-35A2, and actin-1 mRNA. For human THP-1 cells, we quantified the expression of catalase, MnSOD, CuZnSOD, SOD-2, glutathione peroxidase (GPX)-1―4, and GAPDH mRNA. Sequences for the primers used are shown in Table 1.

### 2.11. Statistical Analysis

The locomotion, brood size, and length measurement data were observed to be non-normally distributed using the Shapiro–Wilk test. A survival plot or Kaplan–Meier plot was constructed to evaluate the effects of the different concentrations of SWCNTs−COOH on the lifespan or ageing of the nematodes. One-way ANOVA was used to determine the significant difference of each the concentrations in comparison with the control. The survival plot (Kaplan–Meier plot) was constructed for the lifespan data using GraphPad Prism 6 (San Diego, CA, USA). The nonparametric Kruskal–Wallis test was used to determine the significance of each of the days. Days with significance were chosen and tested further. Each of the concentrations from the chosen days was compared with the control using the Mann–Whitney U test. All statistical analyses were carried out using SPSS version 23 (International Business Machines Corp., New York, NY, USA). In these analyses, *p* < 0.05 represented a statistically significant result.

## 3. Results and Discussion

### 3.1. Characterization of SWCNTs−COOH

**Figure 1:**
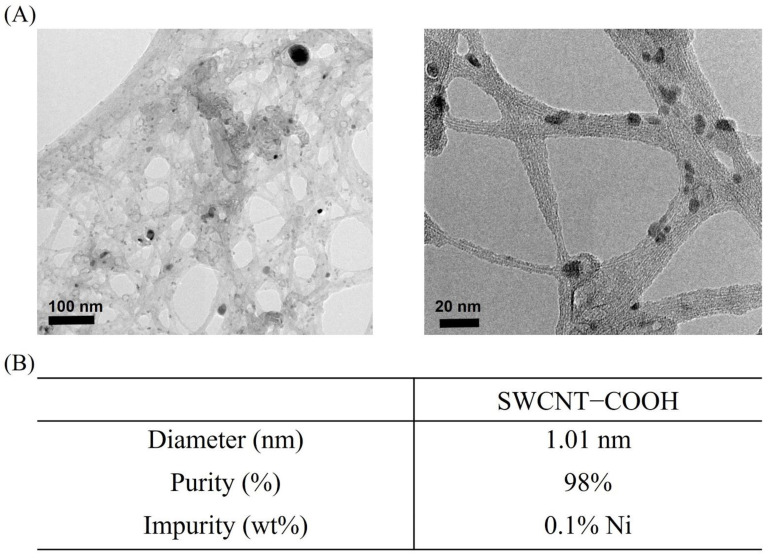
Physiochemical characterization of SWCNTs−COOH in K-medium. (**A**) TEM images of SWCNTs−COOH dispersed in the K-medium with the observed presence of amorphous carbon. (**B**) Summarized properties of the SWCNTs−COOH.

The K-medium is a solution with high ionic strength and, therefore, the dispersion of SWCNTs−COOH in this solution is important when considering their bioavailability for toxicity testing. The SWCNTs−COOH slightly aggregated in the solution, as observed in the TEM images (Figure 1A), and formed bundles. However, effective sonication before every use minimized the aggregation. The average diameter of the resulting SWCNTs−COOH was 1.1 nm, which was consistent with the diameter provided by the manufacturer (Figure 1B). An impurity of 0.1% Ni was detected using EDS. FTIR was used to identify the –COOH functional group of the SWCNTs−COOH sample from our previous paper [21]. According to the results, the existence of C=O was observed to stretch at ~1741 cm^−1^ while C–O stretched at ~1200 cm^−1^, which confirmed the presence of a carboxyl moiety. The length of this SWCNT sample was 1.1 ± 0.6 μm (mean ± standard deviation) according to our prior work [21].

### 3.2. Part I: Caenorhabditis elegans (C. elegans) Animal Model Experiment

#### 3.2.1. Lethal Effects of SWCNTs−COOH

**Figure 2:**
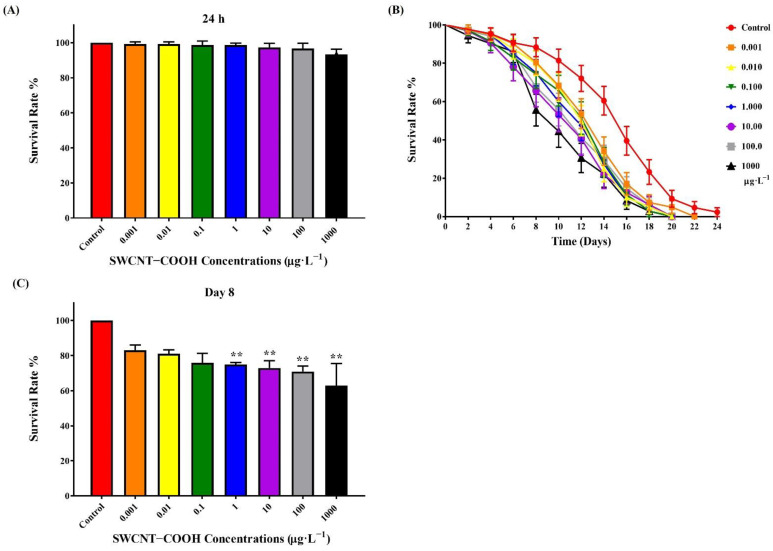
(**A**) Effects of SWCNTs−COOH on the lethality of *C. elegans* after 24 h of exposure. Effects of SWCNTs−COOH on the lifespan of *C. elegans* after 24 days (**B**) and 8 days (**C**) exposure. ** *p* < 0.01 as compared with the control.

The lethality of age-synchronized L4 worms was investigated after exposure to SWCNTs−COOH samples that were serially diluted for 24 h. In comparison with the control group, there were no significantly lethal effects with any of the SWCNTs−COOH concentrations (0.001–1000 μg·L^−1^) (Figure 2A). Few studies have focused on the toxicity of SWCNTs−COOH in *C. elegans.* Treatment with pristine SWCNTs and the amide variant of functionalized SWCNTs (1.0, 100, 250, and 500 μg·mL^−1^) for 48 h was shown to have no significant difference in terms of lethal effects for *C. elegans* as compared with a control group [43]. In other in-vivo models, treatment with SWCNTs−COOH at concentrations of 1.0, 10, 50, 100, or 200 mg·L^−1^ for 72 h significantly reduced the survival of zebrafish (*Danio rerio*) embryos [47]. SWCNTs−COOH concentrations of 0.25, 0.5 and 0.75 mg kg^−1^ body weight induced hepatotoxicity in mice through activation of the oxidative stress mechanism [48]. Arndt et al. (2013) identified no significant lethal effects following the treatment of daphnids with 10 or 50 ppm SWCNTs−COOH for 48 h [22].

#### 3.2.2. Observed Reduced Lifespan of *C. elegans* after Exposure of the Nematodes to SWCNTs−COOH

The toxicological endpoint lifespan was investigated in this study over a duration of 24 days (Figure 2B). The lifespan of *C. elegans* was significantly reduced (75–63%) beginning at 8 days post ≥1 μg·L^−1^ SWCNTs−COOH treatment as compared with the control group (Figure 2C). Our result is similar to that of a previous study, indicating that treatment with 500 μg·mL^−1^ of amide-functionalized SWCNTs also resulted in a decreased lifespan for *C. elegans* (7.8 days vs. 14.3 days in the control) [43]. In contrast, cysteine-functionalized SWCNTs had no significant effects on the lifespan of nematodes [44]. The amide-functionalized SWCNTs were efficiently taken up by *C. elegans*, causing toxic accumulation [43]. Regulation of the translocation and distribution of nanomaterials in both the primary and secondary targeted organs by a biological barrier may affect both the survival and lifespan of *C. elegans* [40]. Carboxyl modification prevented the toxicity of MWCNTs on primary and secondary target organs, but MWCNTs-COOH also persisted in primary target organs [41]. SWCNTs−COOH induced oxidative stress, triggered G2/M phase arrest, increased cell membrane damage, and eventually caused apoptosis [49]. Additionally, functionalized SWCNTs may decrease DAF-16 translocation to the nucleus, which would result in the suppression of the downstream signaling pathway, reducing the lifespan of *C. elegans* [43]. Taken together, these findings reveal that treatment with ≥1 μg·L^−1^ SWCNTs−COOH for 8 days reduces the lifespan of *C. elegans*, an effect that was sustained for 24 days.

#### 3.2.3. Observed Reduced Growth of *C. elegans* after the Exposure of Nematodes to SWCNTs−COOH

**Figure 3:**
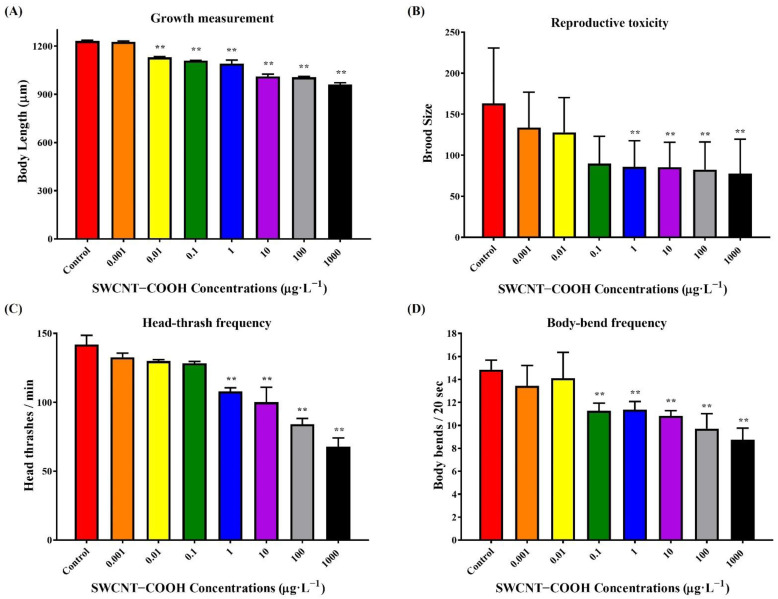
Effects of SWCNTs−COOH on the body length (**A**), brood size (**B**), head thrashing (**C**), and body bending (**D**) of *C. elegans* after 24 h of exposure. ** *p* < 0.01 as compared with control.

Body length is an important manifestation of growth and development in *C. elegans*. To investigate the adverse effect of SWCNTs−COOH on growth, *C. elegans* was exposed to various concentrations of SWCNTs−COOH for 24 h, and then nematodes were transferred to new NGM plates without exposure to SWCNTs−COOH and incubated for 48 h until reaching the L4 stage. As shown in Figure 3A, body length in the control group was 1230 μm, and this was significantly decreased by 0.91-fold (1130 μm) compared with the control group following exposure to a concentration of 0.01 μg·L^−1^ SWCNTs−COOH. The treatment group that was exposed to 1000 μg·L^−1^ SWCNTs−COOH showed the most negative effects. Body length decreased by 0.72-fold (961 μm) compared with the control group. Arndt et al. (2013) found that daphnids exposed to concentrations of 10 and 50 ppm were 7.9% and 17.7% smaller in size than the control group, respectively, and SWCNTs−COOH was shown to be more toxic than SWCNTs, SWCNTs-CONH_2_, and SWCNTs-PEG to the growth of daphnids [22]. Chen et al. (2013) also found a dose-dependent decrease in the body length of *C. elegans* with an increasing concentration of amide-functionalized SWCNTs after 48 h exposure. Furthermore, *C. elegans* was able to recover from amide-functionalized SWCNTs-induced growth retardation following exposure [43]. Nevertheless, SWCNTs−COOH was observed to retard the growth of *C. elegans* at higher concentrations, and the effect was not reversible even after termination of the exposure.

#### 3.2.4. Adverse Effect of SWCNTs−COOH on the Reproduction of *C. elegans*

To examine whether exposure to SWCNTs−COOH affected the reproduction of *C. elegans*, we assayed the brood size after exposure to different concentrations of SWCNTs−COOH (Figure 3B). The result showed that exposure to 1–1000 μg·L^−1^ of SWCNTs−COOH significantly decreased the brood size of *C. elegans* compared with the control group. A total of 163 progenies were produced by the control group, a significant decrease of 0.47-fold (78 progenies) at a concentration of 1000 μg·L^−1^ SWCNTs−COOH compared with the control groups. This corroborates the possibility that a reduction in lifespan might be the resulting effect of penetration of the primary targeted organ ≥1 μg·L^−1^ SWCNTs−COOH, thus affecting the reproductive organs of *C. elegans* which are classified as secondary targeted organs [40,41]. Daphnid reproduction was significantly decreased by 24.6% and 58.7% after chronic exposure to 10 and 50 ppm of SWCNTs−COOH nanomaterials, respectively. In addition, SWCNTs−COOH was found to be more toxic to daphnid reproduction than SWCNTs, SWCNTs-CONH_2_, and SWCNTs-PEG [22]. Similarly, exposure to 500 μg·mL^−1^ of amide-functionalized SWCNTs could cause a decrease in the daily brood size production of *C. elegans* with an average of 43 progenies in all exposed worms [43]. Male albino mice at the age of 7 weeks that underwent continuous exposure to SWCNTs−COOH at a concentration of 4 mg kg^−1^ body weight (b.w.)/twice a week for 5 weeks had decreased epididymis sperm counts, sperm motility, and sperm viability compared with those in a control group [50]. In contrast, long-term (250 μg·L^−1^) and short-term (50, 100, and 250 μg·L^−1^) exposure to cysteine-functionalized SWCNTs had no significant effects on the reproduction of *C. elegans* [44]. The SWCNTs−COOH also accelerated the hatching of zebrafish embryos after 72 h of exposure (acute exposure) [47]. Therefore, the toxicity of SWCNTs−COOH is related to the brood size of the exposed organism.

#### 3.2.5. Effects of SWCNTs−COOH on the Locomotion Behavior of *C. elegans*

The present study used the head thrashing and body bending of *C. elegans* to assess the neurological toxicity effect based on various concentrations of SWCNTs−COOH (Figure 3C,D). Exposure to ≥1 μg·L^−1^ of SWCNTs−COOH significantly decreased head thrashing in *C. elegans* compared with the control group. Compared with the control group (141.9 head thrashes per minute), head thrashing in *C. elegans* decreased by 23.93%, 29.43%, 40.71%, and 52.20% when the SWCNTs−COOH concentration was 1, 10, 100, and 1000 μg·L^−1^, respectively (Figure 3C). Similarly, SWCNTs−COOH significantly decreased body bending in *C. elegans* at concentrations above 0.1 μg·L^−1^. When the SWCNTs−COOH concentration was 0.1, 1, 10, 100, and 1000 μg·L^−1^, the body bending of *C. elegans* decreased by 24.13%, 23.46%, 27.16%, 34.57%, and 41.08%, respectively, compared with the control group (14.85 body bends per 20 s) (Figure 3D). In contrast, cysteine-functionalized SWCNTs had no significant effects on the movement or other aberrant activities of nematodes [44]. Regarding the toxicity of MWCNT, PEGylated MWCNTs reduced the toxicity effect on the brood size and neurobehavioral activity of nematodes in comparison with pristine MWCNTs [40]. Nouara et al. (2013) found that nonfunctionalized-MWCNTs damaged the intestines of *C. elegans* at concentrations above 0.1 μg·L^−1^ and the functions of neurons and reproductive organs at concentrations above 0.001 μg·L^−1^. However, MWCNTs-COOH had no significant effects on the primary and secondary targeted organs at concentrations of less than 100 μg·L^−1^ [41]. The neurons may be secondary targeted organs for CNT toxicants in *C. elegans* [40,41,42]. Locomotion behaviors, including head thrashing, body bending, basic movements, and locomotion rate, are relatively sensitive endpoints for neurotoxicity assessment in *C. elegans.* The combination of previous studies with our findings indicates that the surface functionalization of nanomaterials, such as SWCNTs, may affect the toxicity of *C. elegans*. The potential toxicity of SWCNTs−COOH can be attributed to the damaging effect of the nanomaterials on the reproductive organs and neurons of the nematodes, which may reduce longevity. However, the biological barrier of *C. elegans* may prevent the toxicity effect on secondary targeted organs at low SWCNTs−COOH concentrations.

#### 3.2.6. Effects of SWCNTs−COOH on the Intracellular ROS Generation and Localization of Nematodes

**Figure 4:**
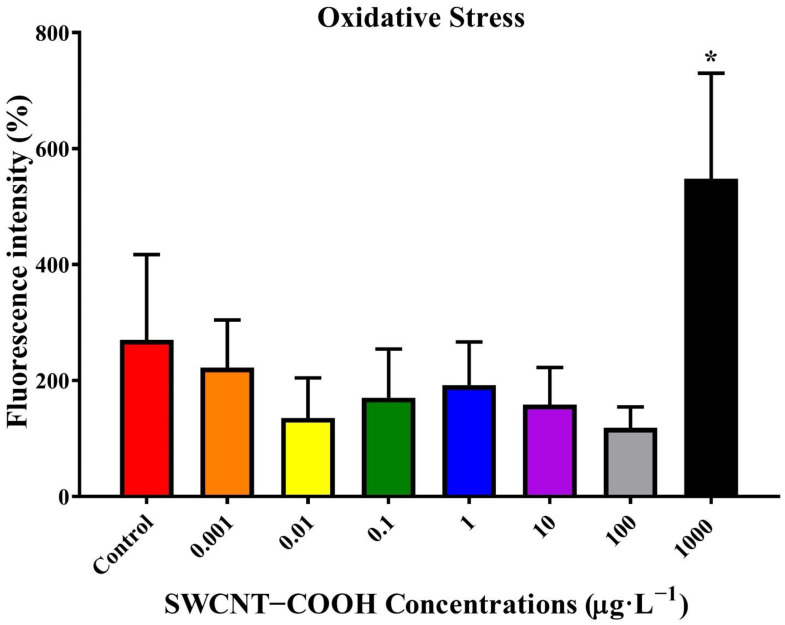
Effects of SWCNTs−COOH on *C. elegans* ROS generation after 8 days of exposure. * *p* < 0.05 as compared with the control.

**Figure 5:**
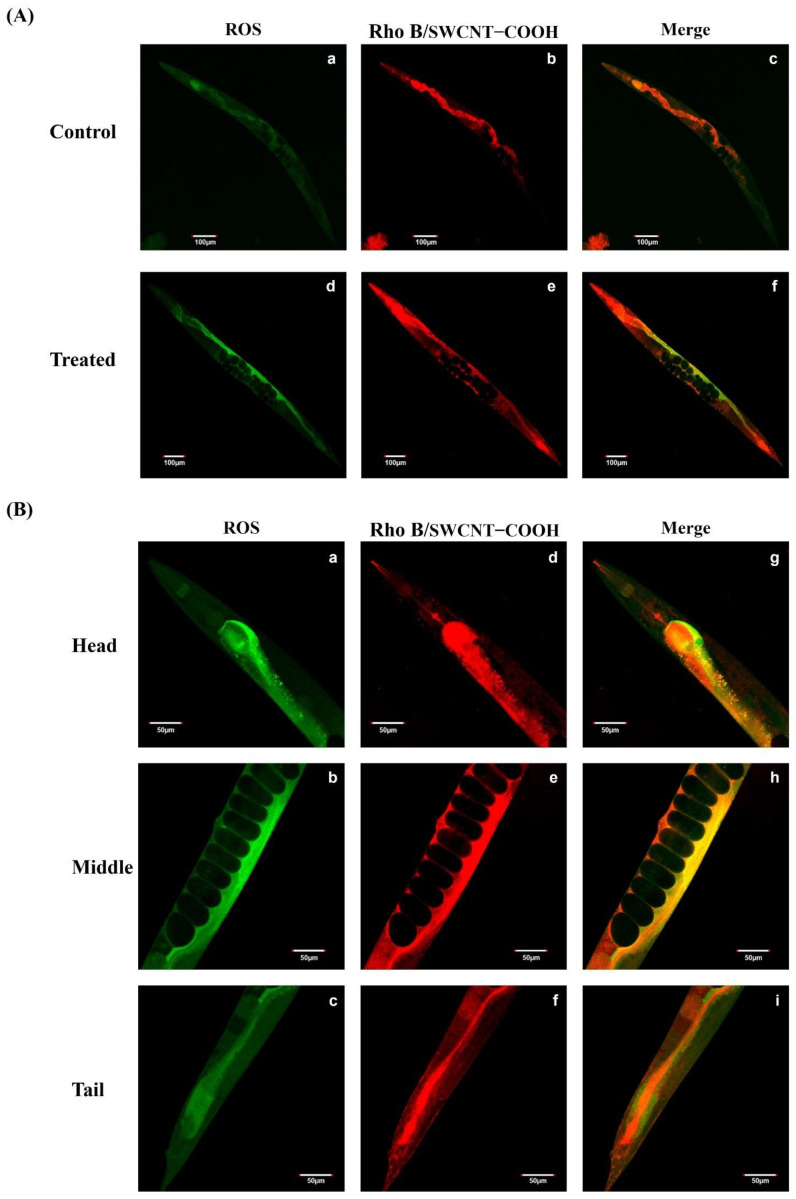
(**A**) Laser-scanning confocal microscope images of *C. elegans* exposed to Rhodamine B-conjugated SWCNTs−COOH (red) and ROS staining dye (green). The images are divided into the merge, Rho B/SWCNTs−COOH only and ROS only for the control (a–c) and the treated worms (d–f). (**B**) Effects of SWCNTs−COOH on *C. elegans* ROS localization after 8 days of exposure. A closer view of the laser-scanning confocal microscope images of *C. elegans* focusing on the head, middle, and tail regions for the ROS only (a–c), RhoB/SWCNTs−COOH only (d–f), and merged (g–i) groups.

This study found that the lifespan of *C. elegans* was significantly reduced beginning at 8 days post ≥1 μg·L^−1^ SWCNTs−COOH treatment (Figure 2C). An investigation of adverse effects addressed the generation and distribution of ROS and the potential mechanisms that occur after exposure of the nematodes to SWCNTs−COOH. As shown in Figure 4, there was no significant effects concerning ROS generation after 8 days of exposure to SWCNTs−COOH at all concentrations, except for 1000 μg·L^−1^ SWCNTs−COOH. In comparison with the control group, the immunofluorescence exam also found that exposure to 1000 μg·L^−1^ SWCNTs−COOH increased ROS generation in the head, middle, and tail regions of *C. elegans* after 8 days (Figure 5A,B).

#### 3.2.7. Upregulation of Antioxidant Gene Expression in SWCNTs−COOH-Treated Nematodes

**Figure 6:**
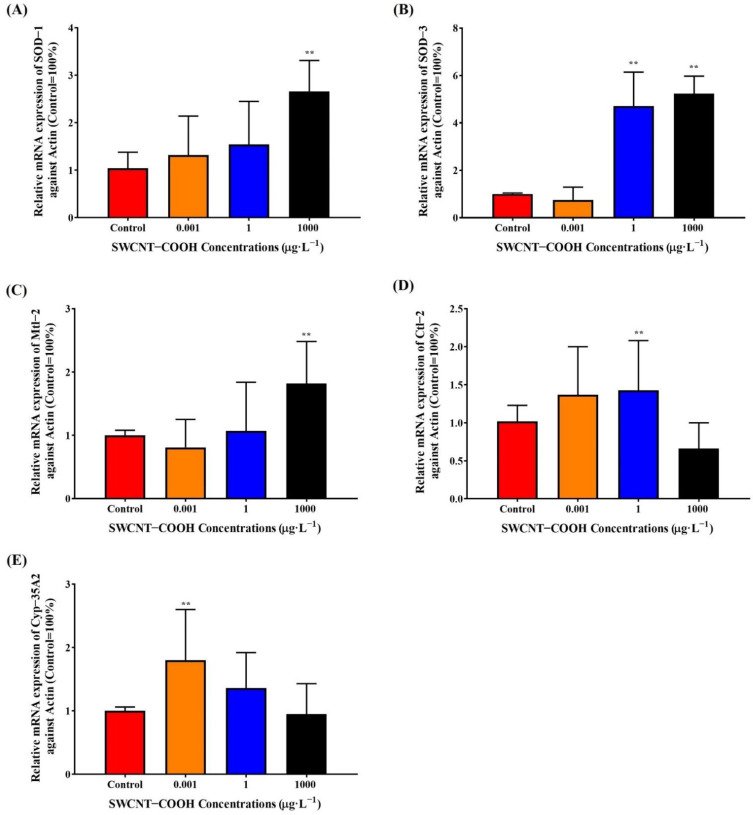
Effects of SWCNTs−COOH on the mRNA expression of SOD-1 (**A**), SOD-3 (**B**), mtl-2 (**C**), ctl-2 (**D**), and cyp-35A2 (**E**) in *C. elegans* after 8 days of exposure. ** *p* < 0.01 as compared with the control.

To investigate the possible toxicity mechanism of SWCNTs−COOH, *C. elegans* was exposed to various concentrations of SWCNTs−COOH at the adult stage for 8 days, and then the expression of SOD-1, SOD-3, CTL-2, CYP-35A2, and mtl-2 mRNA was determined (Figure 6). Real-time PCR analysis showed that exposure to 1000 μg·L^−1^ SWCNTs−COOH significantly increased SOD-1, SOD-3, and mtl-2 mRNA expression; 1 μg·L^−1^ SWCNTs−COOH significantly increased SOD-3 and CTL-2 mRNA expression; and 0.001 μg·L^−1^ SWCNTs−COOH significantly increased CYP-35A2 mRNA expression in *C. elegans* compared with the control group. SOD-1 encodes copper superoxide dismutase and SOD-3 encodes an iron/manganese superoxide dismutase that is localized in the cytosol and/or mitochondrial matrix, which might protect against oxidative stress, regulate the brood size, vulval development, and lifespan [51,52]. CTL-2 has catalase and peroxidase activity, which is required for antioxidant production, a normal lifespan, the egg-laying capacity, and peroxisomal morphology. CYP-35A2 encodes cytochrome P450s and the NADPH-dependent monooxygenasesone of *C. elegans*, which metabolize endogenous compounds (e.g., fatty acids and lipid signaling molecules) and exogenous compounds (e.g., xenobiotic chemicals). In addition, mtl-2 plays roles in stress adaptation, homeostasis, and metal detoxification and might regulate the growth and fertility of *C. elegans* [51]. Our findings reveal that SWCNTs−COOH could trigger a protective response to oxidative damage by increasing the resistance to chronic oxidative stress.

In our previous studies [36,37,38,39], the engineered nanomaterials (ENMs) of silica nanoparticles, including SiO_2_ and NH_2_-SiO_2_, and graphene oxide as well as PM2.5 caused reproductive toxicity, a disruption of neurobehavior, and a shortened longevity for the nematodes. For the ambient PM2.5 level, both low levels of PM2.5 from rural areas and high levels of PM2.5 from heavy traffic emissions in urban areas induced the toxic effects on reproduction, delayed locomotion, and shortened the lifespan in wild-type *C. elegans* models [36,39]. In addition to PM2.5, ENMs were treated and tested in the worms. Based on the findings from our previous reports [37,38] and the present study, the carbon-based ENMs seem to cause greater toxicity than the silica-based ENMs. The carbon-based ENMs have been shown to cause toxic effects at low dosages, for example, graphene oxide at ppt levels [37] and SWCNTs−COOH at ppb levels (in the present study). This is compared with silica-based ENMs, which induce the toxicity of *C. elegans* at high doses, for example, SiO_2_ and NH_2_-SiO_2_ at 1000-fold ppm levels [38]. Graphene oxide nanoparticles at a dose of 0.001 μg·L^−1^ significantly induced oxidative stress (activated gene expression of SOD-1, SOD-3, and CTL-2) to a greater extent than an untreated control [37]. In the present study, SWCNTs−COOH was treated in *C. elegans* to activate the gene expression of SOD-3, CTL-2, and CYP-35A2.

### 3.3. Part II: THP-1 Cells In Vitro Model Experiment

#### 3.3.1. Effects of SWCNTs−COOH on Cell Viability and ROS Generation in THP-1 Cells

**Figure 7:**
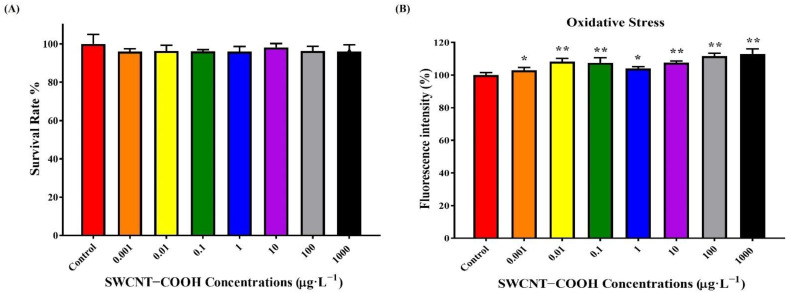
(**A**) Effects of SWCNTs−COOH on the viability of THP-1 cells after 48 h of exposure. (**B**) Effects of SWCNTs−COOH on ROS generation in THP-1 cells after 6 h of exposure. * *p* < 0.05; ** *p* < 0.01 as compared with the control.

Next, we used human monocyte THP-1 cells to explore whether the findings observed in worms were conserved in vertebrates. THP-1 cells were exposed to various concentrations of SWCNTs−COOH for 48 h and the viability of THP-1 cells was measured by the Alamar Blue bioassay, as shown in Figure 7. In comparison with the control group, the SWCNTs−COOH sample concentrations (0.001–1000 μg·L^−1^) had no significant effects on the THP-1 cell viability (Figure 7A). However, 6 h of exposure to 0.001–1000 μg·L^−1^ SWCNTs−COOH significantly induced ROS generation in THP-1 cells, as compared with the control group (Figure 7B).

#### 3.3.2. Alteration of Antioxidant Gene Expression in THP-1 Cells following Treatment with SWCNTs−COOH

**Figure 8:**
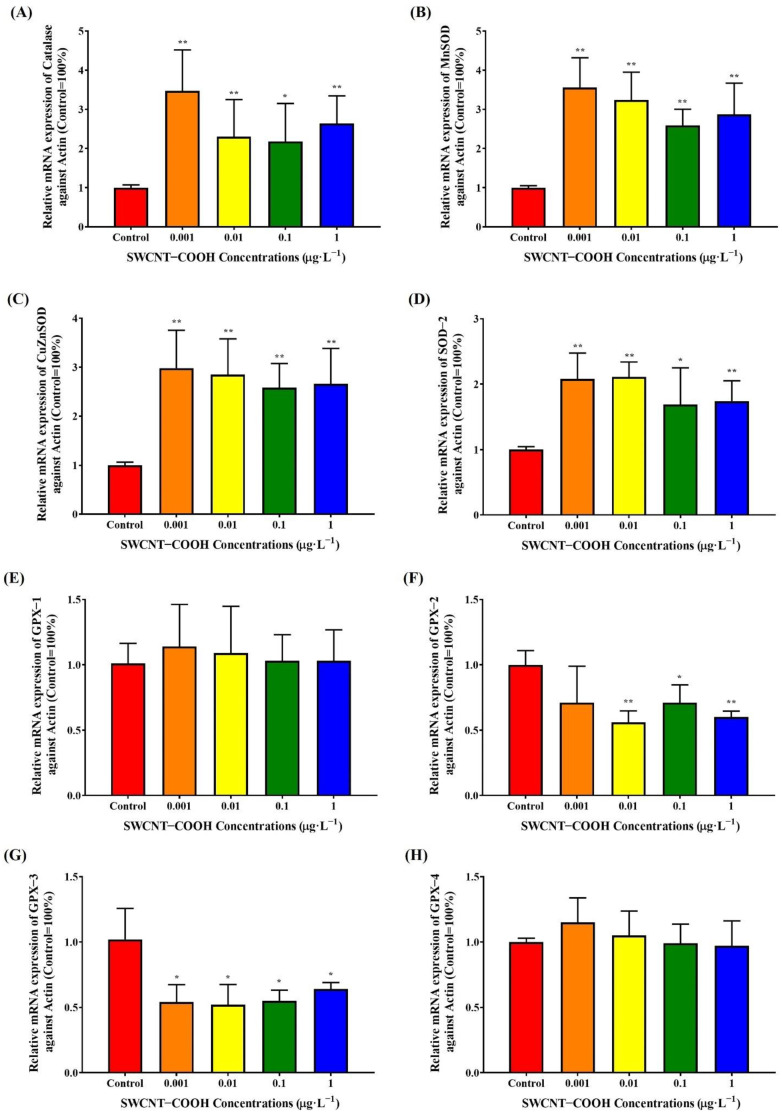
Effects of SWCNTs−COOH on the mRNA expression of catalase (**A**), MnSOD (**B**), CuZnSOD (**C**), SOD-2 (**D**), GPX-1 (**E**), GPX-2 (**F**), GPX-3 (**G**), and GPX-4 (**H**) in THP-1 cells after 48 h of exposure. * *p* < 0.05; ** *p* < 0.01 as compared with the control.

In addition to SOD and catalase, GPX is considered the classical antioxidant enzyme. Therefore, we investigated the mRNA expression of SOD, catalase, and GPX in THP-1 cells after exposure to 0.001–1 μg·L^−1^ of SWCNTs−COOH for 48 h. Real-time PCR analysis showed that treatment with SWCNTs−COOH significantly increased the mRNA expression of catalase, MnSOD, CuZnSOD, and SOD-2 in THP-1 cells compared with the control group (Figure 8A–D). The results were similar to findings for *C. elegans*. However, treatment with 0.01–1000 μg·L^−1^ SWCNTs−COOH significantly decreased GPX-2 and GPX-3 mRNA expression in THP-1 cells compared with the control group (Figure 8E,H). These findings reveal that a low concentration of SWCNTs−COOH might induce ROS generation and disrupt the protective response to oxidative damage in THP-1 cells. Ye et al. (2011) also demonstrated that a low concentration of SWCNTs−COOH (0.1 μg·L^−1^) accumulated mainly in the cytoplasm and was cytotoxic to human primary monocytes, causing increased cell membrane damage, triggering G2/M phase arrest, inducing an inflammatory response through oxidative stress and NF-κB activation, and eventually causing apoptosis [49]. Furthermore, background-level SWCNTs−COOH induced antioxidant gene expression in nematodes that was similar to high homologous gene expression with human cells, indicating that the effects produced by SWCNTs−COOH may be conserved across different species.

## 4. Conclusions

The present study revealed that exposure to a low dose of SWCNTs−COOH (0.001 or 1.00 μg·L^−1^) induced ROS generation and disrupted antioxidant gene expression in human monocytes THP-1 cells. Similar nanotoxic effects were also found in *C. elegans*, where treatment with 0.001 or 1.00 μg·L^−1^ SWCNTs−COOH disrupted the expression of antioxidant genes, including SOD-1, SOD-3, and ctl-2. SWCNTs−COOH (≥1 μg·L^−1^) was also associated with adverse effects on lifespan, growth, reproduction, locomotion behavior, and ROS generation in *C. elegans*. Our data, which was collected using in-vitro cultured THP-1 cells and an in-vivo *C. elegans* model, reveal that a background level of SWCNTs−COOH may exert nanomaterial toxicity. Further in-depth studies of the molecular mechanism underlying the effects of SWCNTs−COOH using mammalian animals and clinical trials are required.

## Figures and Tables

**Table 1:** Primer sequences.

Gene Code	Forward Primer (5′ to 3′)	Reverse Primer (5′ to 3′)
*C. elegans*		
SOD-1	ACGCTTTACGGTCCAAACAC	ATTCAGAGACGCGATTCAGG
SOD-3	TTGAAGATCGCCACCTGTGCAAAC	ATGGACATAGTCTGGGCGGACATT
mtl-2	AAAAGGCCTACCAGCAGACA	GCAGCAGTATTGCTCACAGC
ctl-2	TCCGTGACCCTATCCACTTC	TGGGATCCGTATCCATTCAT
cyp-35A2	TGCTGGTAAATATGCGGACA	ACACCATTGGTTGCCAATTT
Actin-1	AGAAGAGCACCCAGTCCTCC	GAAGCGTAGAGGGAGAGGAC
THP-1		
Human CAT	CTGGGACTTCTGGAGCCTAC	CAACTGGGATGAGAGGGTAG
Human MnSOD	AGAAGTACCAGGAGGVGTTG	AGTGTCCCCGTTCCTTATTG
Human CuZnSOD	AGGGCATCATCAATTTCGAG	CCATCTTTGTCAGCAGTCAC
Human SOD2	TGCACTGAAGTTCAATGGTGG	CTTCCAGCAACTCCCCTTTG
Human Gpx1	GAAGTGCGAGGTGAACGGTG	GGGATCAACAGGACCAGCAC
Human Gpx2	AGATGTGGCCTGGAACTTTG	CATTCTGTGAAGGCCCAGAG
Human Gpx3	GAAAGGGGATGTCAATGGAG	ATGAGACGGCCTTCAGTTAC
Human Gpx4	CCAGTGAGGCAAGACCGAAG	CAGCCGTTCTTGTCGATGAG
Human GAPDH	TGGACCTGACCTGCCGTCTA	CCCTGTTGCTGTAGCCAAATTC

## Data Availability

The original contributions presented in the study are included in the article.
